# Supplementary figures and images for: RNA-Seq Provides Insights into VEGF-Induced Signaling in Human Retinal Microvascular Endothelial Cells: Implications in Retinopathy of Prematurity
Source: Int J Mol Sci. 2022 Jul 1;23(13):7354. doi: 10.3390/ijms23137354 (PMC9266443; doi:10.3390/ijms23137354)

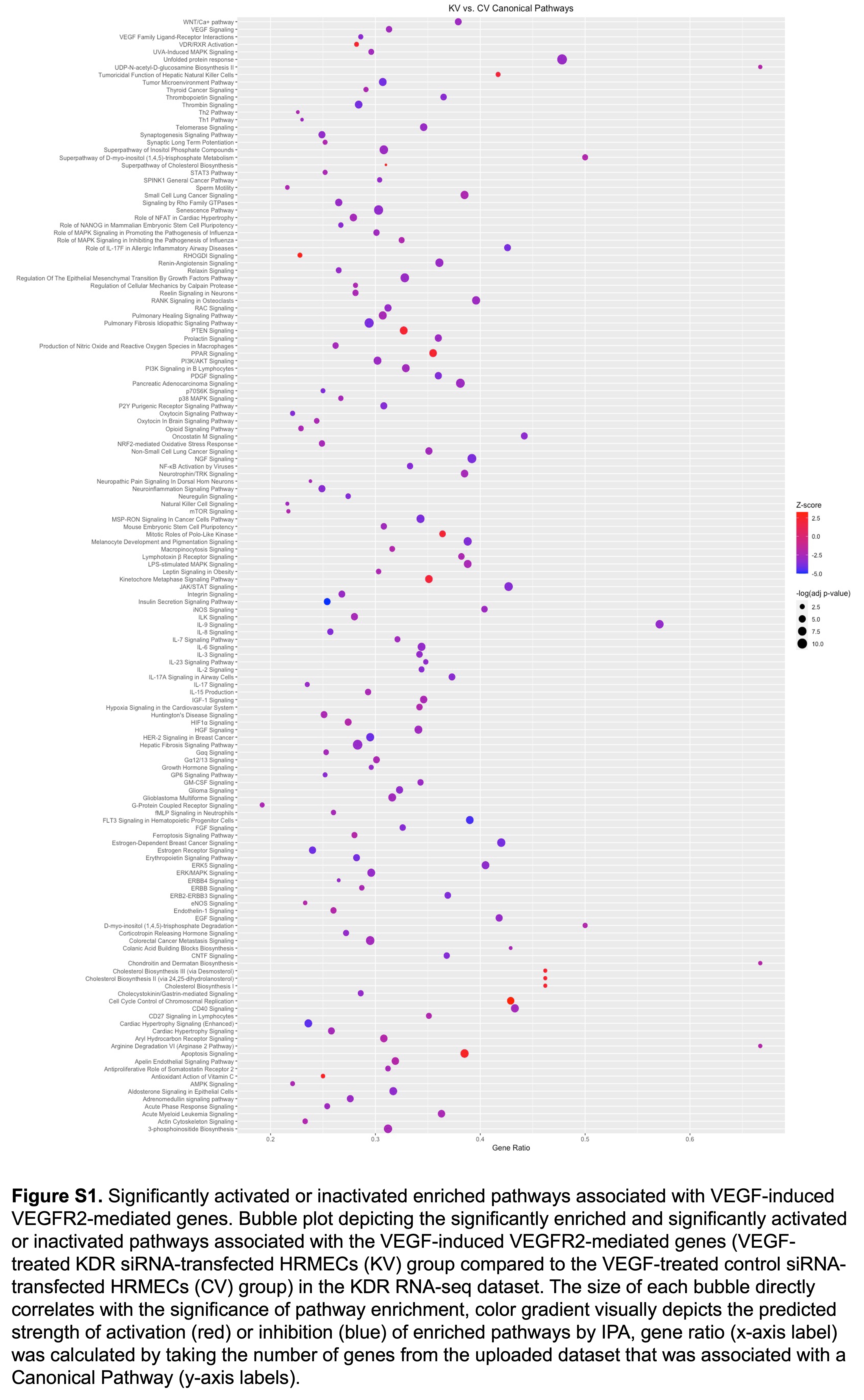

Supplement: Supplementary file 1 [file ijms-23-07354-s001.zip › FigureS1.jpg]

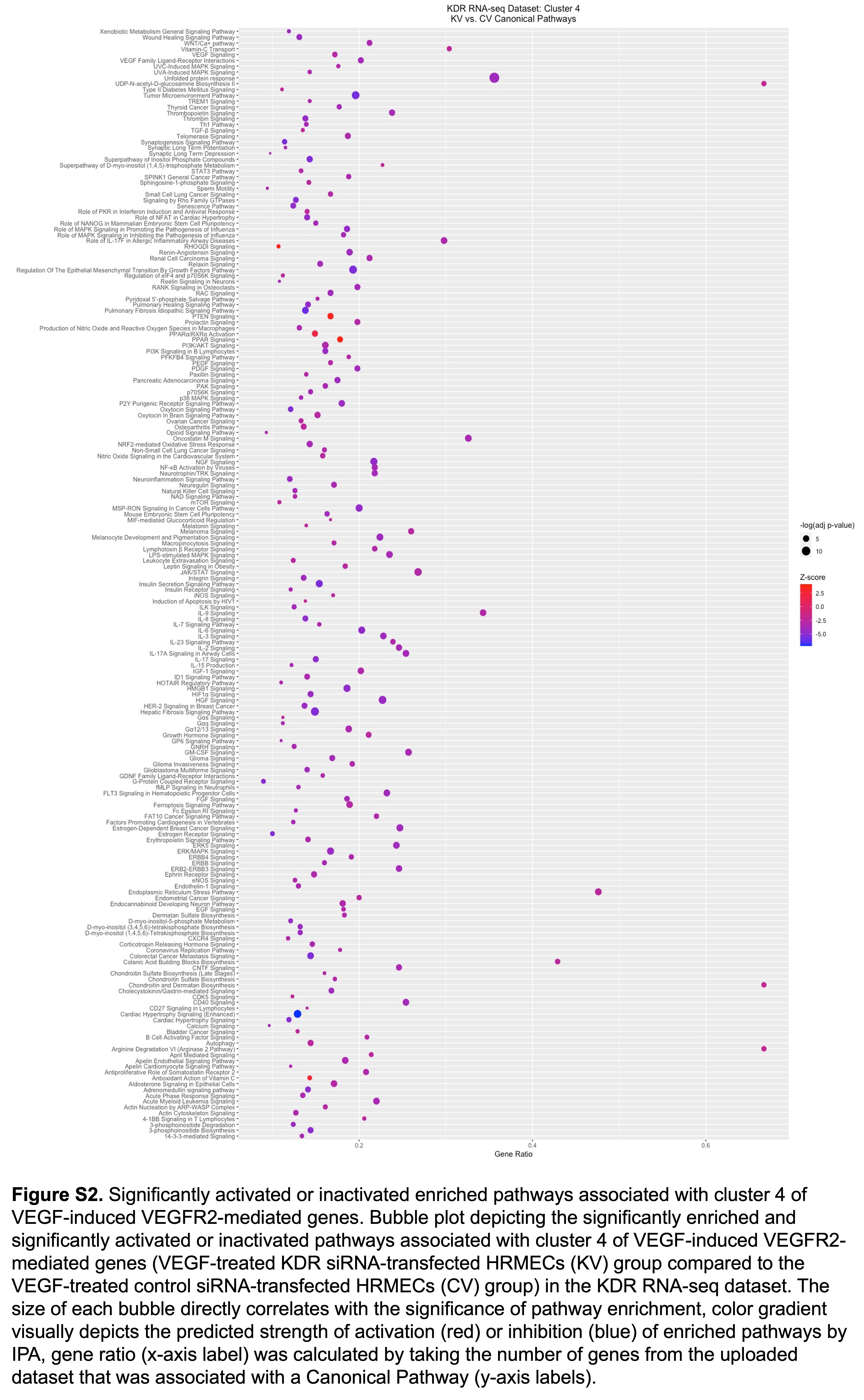

Supplement: Supplementary file 1 [file ijms-23-07354-s001.zip › FigureS2.jpg]

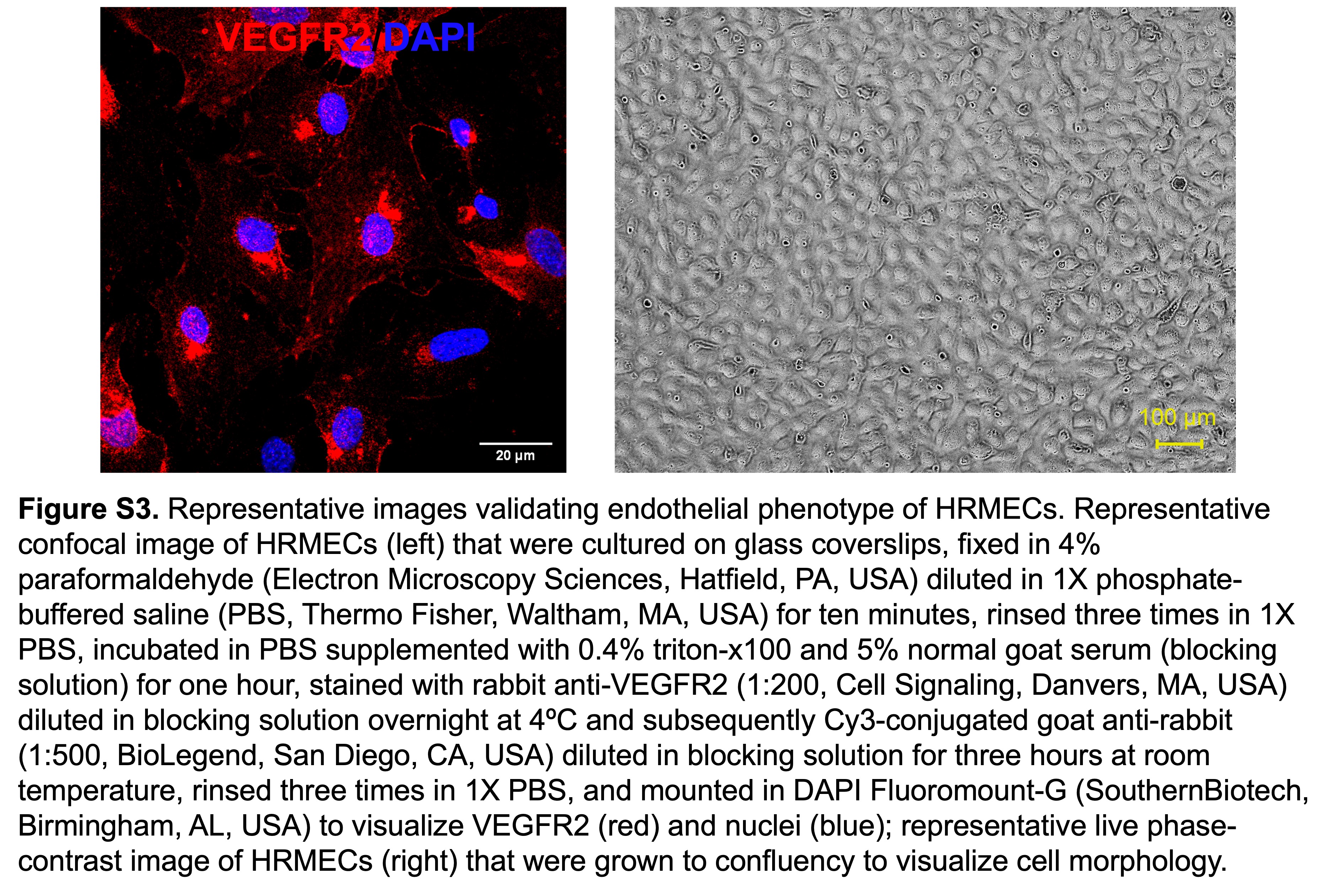

Supplement: Supplementary file 1 [file ijms-23-07354-s001.zip › FigureS3.jpg]
